# Editorial: Inflammation, Metabolism and Epigenetics in Valvular Heart Disease

**DOI:** 10.3389/fcvm.2022.880015

**Published:** 2022-04-11

**Authors:** Eva Jover, Ashton Faulkner, Paolo Madeddu, Natalia López-Andrés

**Affiliations:** ^1^Cardiovascular Translational Research, Navarrabiomed, Complejo Hospitalario de Navarra (CHN), Universidad Pública de Navarra (UPNA), Instituto de Investigación Sanitaria de Navarra (IdiSNA), Pamplona, Spain; ^2^School of Biochemistry, University of Bristol, Bristol, United Kingdom; ^3^Bristol Medical School, Translational Health Sciences, University of Bristol, Bristol, United Kingdom

**Keywords:** valvular heart disease, inflammation, metabolism, sex-differences, epigenetics, non-coding RNA, biomarkers

The prevalence of valvular heart disease (VHD) within the elderly increases from 2.8% in individuals aged 60–74 years to 13.1% in the over 75's. Moreover, with a continually aging population, VHD is predicted to become the forthcoming cardiac epidemic (1). Owing to the severe disease burden, VHD is a major source of morbidity and mortality. More than three decades of research have revealed that highly multifactorial and complex cues trigger molecular, cellular and interstitial events. Valve resident cells, namely valve interstitial (VIC) and endothelial cells (VEC), are instrumental to the VHD pathogenesis (2). Although valve replacement remains the primary therapeutic choice, current limitations entail expensive treatments, hospital readmission and reintervention. Accumulating evidence suggests that inflammation from different sources (e.g., atherosclerosis, autoimmune diseases, diabetes) might be mechanistically relevant during early and late stages of VHD. This Research Topic “*Inflammation, Metabolism and Epigenetics in Valvular Heart Disease*” includes four articles gathering an interdisciplinary overview of the contribution of inflammation to disease progression and prognosis, and explores the interplay among inflammation, metabolism, epigenetics and sex-related mechanisms underpinning VHD.

As reviewed by Ferrari and Pesce, the endothelial insult exerted by different risk factors may initiate and exacerbate the damage of the aortic valve. Interleukins, oxidative stress and apoptotic pathways promote a myriad of aberrant events. Inflammatory stimuli may underpin metabolic switches in quiescent VIC and VEC to accommodate the energetic requirements of divergent pathologic phenotypes, including differentiation into myofibroblast, osteoblast or pericyte-like phenotypes. It is now becoming evident that metabolites can raise regulatory effects on a vast array of pharmacologically targetable epigenetic regulators and vice versa (Ferrari and Pesce). Methylation and acetylation status or the non-coding RNAs can regulate NOTCH1 potentially impacting the expression of inflammatory and osteogenic genes. The role of miRNAs as biomarkers, therapeutic targets or therapeutic alternatives is additionally discussed. Different miRNome signatures may support specific forms of VHD. Importantly, the authors describe sex-related differences in aortic valve stenosis (AVS) phenotypes. The ideological trend linking calcification to AVS severity might be becoming obsolete in light of the phenotypic presentation of AVS in women. The increasing number of publications and special issues launched by internationally indexed journals, such as *Frontiers in Cardiovascular Medicine*, evidence the growing interest on elucidating sex-related differences in AVS. This exhaustive review prompts to investigate in depth the molecular and metabolic mechanisms underlying AVS to develop new therapeutic opportunities.

Diabetes increases the rate of progression from mild to severe forms, thus being acknowledged as a marker of poor prognosis in AVS. The naïve aortic valve is one of the earliest cardiovascular beds affected by concomitant hyperglycemia and hyperlipidemia, and bioprosthetic valves implanted in diabetic patients are more prone to deterioration. Cecoltan et al. demonstrate that high glucose triggers pro-inflammatory phenotypes and endothelial-to-mesenchymal transition in human VECs engineered on a physiologically relevant extracellular matrix (ECM). Decellularized collagen type I and III-rich ECM derived from porcine aortic roots were re-populated with human VECs and VICs to mimic the valve cell disposition in the naïve fibrosa. Accordingly, this study confers a valuable insight into the contribution of diabetes-related dysfunction of the VEC and offers a novel methodological approach to the repertoire of VHD research models. Moreover, the authors propose the pro-inflammatory endothelin-1 (ET-1) signaling to be potentially targetable to prevent the secretory and pro-inflammatory switch of the VEC, likely contributing to the development of AVS in diabetic patients. They also speculate that high glucose-related up-regulation of ET-1 and interleukin-1β might contribute to the endothelial-to-mesenchymal activation of the VEC, in a similar manner to that observed in diabetic cardiac fibrosis.

Park et al. have systematically reviewed the literature to provide a thorough vision of the actual prevalence and presentation of VHD in patients with autoimmune spondyloarthritis. It is not a new concept that patients with spondyloarthritis develop cardiovascular arrythmias and VHD (e.g., “HLA-B27 associated cardiac disease”). Among more than 500 indexed publications, aortic insufficiency was found to be the most prevalent form of VHD with a hazard ratio of 1.8–2.0 and an increased risk of undergoing elective surgical valve replacement, while after adjusting for age such association was not significant. Other reported abnormalities were mitral valve prolapse, calcification, valve and aorta thickening or aortic root dilatation. Although interesting, preliminary findings are based on heterogeneous, small sample-sized and non-adjusted case-control studies that preclude the drawing of actual associations between spondyloarthritis and cardiac manifestations. Moreover, the SIGN Approach used in this review rates these studies to have a low evidence quality. Indeed, several recent studies did not find significant associations among spondyloarthritis and VHDs. Nevertheless, owing to the autoimmune inflammatory burden and the histological similarities (e.g., resident T cells) reported between the aortic valve attachment site and the articular enthesis, the authors hypothesize a distinctive form of valvular tissue degeneration exists in spondyloarthritis patients leading to different VHD phenotypes. Accordingly, they encourage to systematically reappraise the evidence of VHD in spondyloarthritis.

Lin et al. have prospectively assessed the inflammatory C-reactive protein (CRP) (cut-off 6.5 mg/mL) at hospital discharge to predict the excessive 1-year mortality of acute infective endocarditis (IE) patients. This observational study recruited 343 patients with acute IE who were categorized as low or high-CRP patients at hospital discharge. Mortality rate was consistently higher in high-CRP compared with low-CRP patients after 1-year follow-up with an excess hazard ratio of 4.2 after multivariate analysis. The occurrence of paravalvular abscess was also enhanced in high-CRP patients, whilst no differences were seen at the cumulative rate of re-hospitalization. This study suggests that high levels of CRP at hospital discharge might be a marker of poor prognosis in acute IE patients. As per other forms of cardiovascular disease, researching and elaborating appropriate panels of VHD prognosis markers must be fostered to identify patients at high risk and provide useful management tools.

A better understanding of the inflammatory sources and its contribution to the VHD pathogenesis, as well as the interplay among inflammation, metabolism and epigenetic regulation might open new avenues to identify druggable targets and improve current therapies. It is also clear that sex-related differences also need to be considered in the current and future research of the VHD.

## Author Contributions

All authors listed have made a substantial, direct, and intellectual contribution to the work and approved it for publication.

## Funding

This work was supported by a Sara Borrell grant (CD19/00251) from the Instituto de Salud Carlos III by a Miguel Servet contract CP13/00221 from the Instituto de Salud Carlos 674 III-FEDER, Fondo de Investigaciones Sanitarias (PI18/01875), and by the British Heart Foundation (BHF) Centre for Cardiovascular Regenerative Medicine Award (II)-Centre for Vascular Regeneration (RM/17/3/33381).

## Conflict of Interest

The authors declare that the research was conducted in the absence of any commercial or financial relationships that could be construed as a potential conflict of interest.

## Publisher's Note

All claims expressed in this article are solely those of the authors and do not necessarily represent those of their affiliated organizations, or those of the publisher, the editors and the reviewers. Any product that may be evaluated in this article, or claim that may be made by its manufacturer, is not guaranteed or endorsed by the publisher.
